# Potential Food Application of a Biosurfactant Produced by *Saccharomyces cerevisiae* URM 6670

**DOI:** 10.3389/fbioe.2020.00434

**Published:** 2020-05-07

**Authors:** Beatriz Galdino Ribeiro, Jenyffer M. Campos Guerra, Leonie Asfora Sarubbo

**Affiliations:** ^1^Northeast Biotechnology Network, Federal Rural University of Pernambuco, Recife, Brazil; ^2^Department of Chemical Engineering, Federal University of Pernambuco, Recife, Brazil; ^3^Center of Sciences and Technology, Catholic University of Pernambuco, Recife, Brazil; ^4^Advanced Institute of Technology and Innovation, Recife, Brazil

**Keywords:** agro-industrial waste, antioxidant activity, biosurfactant, food, thermal analysis, yeast

## Abstract

Biosurfactants have aroused considerable interest due to the possibility of acquiring useful products that are tolerant to processing techniques used in industries. Some yeasts synthesize biosurfactants that offer antioxidant activity and thermal resistance and have no risk of toxicity or pathogenicity, demonstrating potential use in food formulations. The aim of the present study was to assess the use of a biosurfactant produced by *Saccharomyces cerevisiae* URM 6670 to replace egg yolk in a cookie formulation. The yeast was grown in a medium containing 1% waste soybean oil and 1% corn steep liquor. The biosurfactant was isolated using a novel method and was structurally characterized using FT-IR, NMR, and GC/FID. Thermal stability was determined using thermogravimetry (TG)/differential scanning calorimetry (DSC) and antioxidant activity was investigated using three methods. Cytotoxicity tests were performed using the MTT assay with mouse fibroblast and macrophage lines. In the final step, the biosurfactant was incorporated into the formulation of a cookie dough replacing egg yolk. The physical properties and texture profile were analyzed before and after baking. The surface and interfacial tensions of the culture medium after the production process were 26.64 ± 0.06 and 9.12 ± 0.04 mN/m, respectively, and the biosurfactant concentration was 5.84 ± 0.17 g/L after isolation. In the structural characterization by NMR and FT-IR, the biosurfactant from *S. cerevisiae* exhibited a glycolipid structure, with the fatty acid profile revealing a high percentage of linoleic acid (50.58%). The thermal analysis demonstrated stability at the industrial application temperature, with the negligible loss of mass at temperatures of up to 200°C. The biosurfactant was non-toxic to the fibroblast and macrophage cell lines, with cell inhibition less than 15%. The incorporation of the biosurfactant into the cookie dough did not alter the physical or physicochemical properties of the product after baking. In the analysis of the texture profile before baking, the substitution of egg yolk with the biosurfactant did not alter the properties of firmness, cohesiveness, or elasticity compared to the standard formulation. Therefore, the biosurfactant produced by *S. cerevisiae* URM 6670 has potential applications in the food industry as a replacement for egg yolk.

## Introduction

Biosurfactants are molecules of biological origin (e.g., microorganisms) capable of reducing the surface and interfacial tensions. The surfactant activity of these natural compounds is due to the existence of both hydrophilic and hydrophobic moieties in the molecular structure (Rane et al., 2017; López-Prieto et al., 2019). Biosurfactants have aroused considerable interest in recent decades due to their advantageous properties over synthetic surfactants, such as high selectivity and biodegradability, stability in a range of environmental conditions (temperature, pH, and salinity), and low toxicity, favoring their application in the recovery of hydrophobic compounds and as emulsifiers in the pharmaceutical, cosmetic, and food industries (Garg and Priyanka, 2018; Felix et al., 2018).

To make the production of biosurfactants economically feasible, it is important to lower the cost of the fermentation media, which account for about 50% of the final cost of the product (Jimoh and Lin, 2020; Lima et al., 2017; Santos et al., 2016). For such, media incorporating agro-industrial waste products have been proposed as substrates to improve the viability of the large-scale production of biosurfactants and make these natural products more competitive (Lima et al., 2020; Sarubbo et al., 2015).

In the food industry, biosurfactants can be used for the cleaning and/or treatment of contact surfaces, acting as antimicrobial and anti-biofilm agents, and can also be incorporated directly into formulations as an additive or ingredient (Nitschke and Silva, 2018; Khanna and Pattnaik, 2019). The use of biosurfactants as food additives is in line with the growing consumer interest in natural, organic, vegan foods, requiring the development of biomolecules with technological properties capable of reducing or even eliminating the use of synthetic surfactants (Jahan et al., 2020).

The emulsification capacity is another a very attractive property of these biomolecules for food products. The terms “biosurfactants” and “bioemulsifiers” are often used interchangeably. However, those that reduce surface tension at the air–water interface are called biosurfactants and those that lead to emulsification are called bioemulsifiers. Biosurfactants very often have emulsifying capacity, but bioemulsifiers do not necessarily reduce surface tension (Mujumdar et al., 2019; Tao et al., 2019).

The incorporation of biosurfactants has been explored in several formulations, leading to the need for the identification of novel surfactant compounds produced by different microorganisms grown with waste products to reduce costs (Akbari et al., 2018; Salek and Euston, 2019). Among the different types of biosurfactants explored, lipopeptides and glycolipids stand out due to their desirable properties for application in the food industry, such as antibacterial and anti-adhesive activity against a variety of species (*Pseudomonas aeruginosa*, *Escherichia coli*, *Bacillus subtilis*, and *Staphylococcus aureus*) (de Freitas Ferreira et al., 2018; Gaur et al., 2019), antioxidant activity (Zouari et al., 2016b; Jamshidi-Aidji et al., 2019), and low cytotoxicity (Balan et al., 2019). Biosurfactants are also effective at solubilizing vegetable oils, stabilizing fats during cooking processes, and improving the organoleptic properties of bread. Biomolecules can be used in ice cream formulations (Pessôa et al., 2019), muffins (as an ingredient to replace baking powder and eggs; Kiran et al., 2017), cookies (for the replacement of synthetic additives; Zouari et al., 2016a) and salad dressings (as an emulsifier; Campos et al., 2019). In farinaceous foods, the use of emulsifiers of microbial origin emerged to reduce the use of currently marketed emulsifiers and improve the rheology of the products (Kieliszek et al., 2017).

The aim of present study was to assess the use of a biosurfactant produced by *Saccharomyces cerevisiae* URM 6670 grown with waste soybean oil and corn steep liquor to replace egg yolk in a cookie formulation.

## Materials and Methods

### Materials

All chemical reagents were of analytical grade. Waste soybean oil came from restaurants in the city of Recife (Brazil) and was used without any further processing. Corn steep liquor was obtained from Corn Products do Brasil (Cabo de Santo, Brazil). The ingredients for the food formulation were obtained from supermarkets in the same city.

### Microorganism

The yeast *S. cerevisiae* URM 6670 was obtained from the culture collection of the Department of Antibiotics of the Federal University of Pernambuco (Brazil) and kept in a yeast mold agar (YMA) medium containing yeast extract (0.3%), D-glucose (1%), peptone (0.5%) and agar (2%), pH 7.0. The growth medium, yeast mold broth (YMB), had the same composition, excluding agar. Transfers were made to fresh agar slants each month to maintain viability. For preparation of the inoculum, the yeast was grown on the solid medium at 27°C for 48–72 h. A loopful of the cream colored young culture in the YMA medium was then transferred to flasks containing 50 mL of YMB, followed by incubation at 28°C for 24 h with shaking at 200 rpm.

### Production and Isolation of Biosurfactant

To produce the biosurfactant, 2% (v/v) inoculum (10^8^ cells/mL) were added to a medium containing (w/v) 1.0% of waste soybean oil and 1.0% of corn steep liquor (pH 6.8) in distilled water. The medium was incubated at 28°C under 150 rpm for 120 h (Selvakumar et al., 2016). The non-centrifuged culture medium, i.e., whole broth obtained after cultivation, was used for the extraction of the biosurfactant. After fermentation, the biosurfactant was isolated using ethyl acetate solvent twice at a 1:4 (v/v) ratio with the non-centrifuged medium. After phase separation, the organic phase was centrifuged (2600 g for 20 min) and filtered. The residual aqueous phase in the organic phase was removed again with the addition of saturated sodium chloride (NaCl) and anhydrous magnesium sulfate (MgSO_4_) and the organic phase was dried at 50°C (Ribeiro et al., 2019).

### Determination of Surface and Interfacial Tension

Changes in surface tension were determined in the cell-free broth obtained by centrifuging the cultures at 35000 rpm for 20 min. Surface tension was determined using a Sigma 700 Tensiometer (KSV Instruments Ltd., Finland) at room temperature. Tensiometers determine the surface tension with the aid of an optimally wettable ring suspended from a precision scale. With this method, the liquid is raised until contact with the surface is registered. The sample is then lowered again so that the film produced beneath the liquid is stretched for the determination of maximum force, which is used to calculate surface tension. The interfacial tension was measured in the same way in relation to n-hexadecane (Silva et al., 2014).

### Characterization of Biosurfactant

#### Nuclear Magnetic Resonance Spectroscopy

NMR experiments were performed with a VNMRS400 spectrometer (Varian, Palo Alto, CA, United States) operating at 400.0 and 100.0 MHz for the ^1^H and ^13^C nucleus, respectively. The biosurfactant was dissolved in CD3OD. The residual signal of the solvent (*δ*_H_ 3.31 ppm) and the signal of the methyl group (*δ*_C_ 49.0 ppm) were used as reference for the chemical shift to ^1^H and ^13^C-NMR spectra, respectively. To assess microbial action, the organic phase of the samples was extracted with CDCl3 and the residual signal of the solvent (*δ*_H_ 7.26 ppm) was used as reference. Spectra were determined with a PFG 5 mm probe, pulse of RF equivalent at 45°, an acquisition time of 3.2 s, delay of 1.0 s and 64 repetitions.

#### Fourier Transform Infrared Spectroscopy

The biosurfactant extract was also characterized by Fourier transform infrared spectroscopy (FTIR, 400 Perkin Elmer). The signals were collected from 400 to 4000 wavenumbers with a resolution of 4 cm^–1^.

#### Determination of Fatty Acid Composition

For determination of the fatty acid profile, the biosurfactant was submitted to the esterification process. For such, a 25-mg sample of surfactant was subjected to reaction with 0.5 mL of a potassium hydroxide solution (KOH) in methanol at 0.5 mol/L under agitation in vortex tube shaker for 2 min. Hexane was then added for the separation of the polar molecule esters and the mixture was subjected to agitation and subsequent centrifugation (4500 rpm for 6 min). For the analysis, the organic phase was collected and filtered through a polytetrafluoroethylene membrane with a porosity of 0.22 μm, followed by analysis in the gas chromatograph with a flame ionization detector (GC/FID; Agilent Technologies 7890A). The analyses were carried out in a gas chromatograph equipped with a DB-5ms capillary column (30 m in length × 250 μm diameter × 0.25 μm). The carrier gas was helium at a flow rate of 1 mL/min. The injector and detector (FID) temperature was 300°C. The oven temperature was increased to 150°C at 1°C/min, held for 4 min, increased to 280°C at 4°C/min, and held for 5 min. The identification of fatty acids was performed using an external standard (FAME Supelco^TM^ mix C4-C24, Bellefonte, PA, United States) and the percentage composition was calculated based on the normalization of peak areas.

### Thermal Analysis of Biosurfactant

Thermal analysis of the isolated biosurfactant was performed by differential scanning calorimetry (DSC) and thermogravimetry (TG) in a simultaneous thermal analyser (STA 449 F3, NETZSCH) using 50 mg of sample. For such, a nitrogen atmosphere was used (50.0 mL/min flow) in successive heating/cooling/heating steps at a rate of 10°C/min, with the temperature ranging from 40 to 400°C, as described by Han et al. (2015).

### Antioxidant Activity of Biosurfactant

#### Total Antioxidant Capacity

The total antioxidant capacity (TAC) of the isolated biosurfactant was determined by adding 1.0 mL of a 600 mM sulfuric acid, sodium phosphate and 4 mM ammonium molybdate solution to 0.1 mL of biosurfactant solution at different concentrations (1.25, 2.5, 5.0, 10.0, 20.0, and 40.0 mg/mL). The solutions were then placed in a 90°C water bath for 90 min. After cooling, 200 μL of each solution were transferred to a microplate (96 wells) and absorbance was measured in an Elisa plate reader (BioTek) at 695 nm, with the TAC of the biosurfactants expressed in relation to the standard of ascorbic acid (1000 μg/mL), the reference antioxidant activity of which was considered equal to 100% (Prieto et al., 1999).

#### Evaluation of the Sequestering Activity of 2,2-Diphenyl-1-Picrylhydrazyl Radical

The evaluation of the antioxidant activity of the biosurfactant by the free radical scavenging method was measured by means of hydrogen donation using the stable radical 2,2-Diphenyl-1-Picrylhydrazyl (DPPH; Brand-Williams et al., 1995). A stock solution of methanolic DPPH (200 μM) was further diluted in methanol to reach UV–VIS absorbance between 0.6 and 0.7 at 517 nm, obtaining the DPPH working solution. Solutions of the biosurfactant (40 μL) at different concentrations (1.25, 2.5, 5.0, 10.0, 20.0, and 40.0 mg/mL) were mixed with DPPH solution (250 μL). After 30 min of incubation in the dark, the absorbances were read at the same wavelength mentioned above. The measurements were carried out in triplicate and the inhibition activities were calculated based on the percentage of DPPH eliminated. The percentage of inhibition (I%) was calculated using the following equation: I% = [(Abs_0_ - Abs_1_)/Abs_0_] × 100, in which Abs_0_ is the control absorbance and Abs_1_ is absorbance in the presence of the biosurfactant. A standard solution of the synthetic antioxidant Trolox (6-hydroxy-2,5,7,8-tetramethychroman-2-carboxylic acid) at a concentration of 10–200 μM was used for the calibration curve.

#### Sequestration of Superoxide Ion

To determine the sequestration of superoxide ion, 50 μL of the biosurfactant solution (1.25, 2.5, 5.0, 10.0, 20.0, and 40.0 mg/mL) diluted in phosphate buffer 150 mmol/L, 200 μL of 65 mmol/L methionine solution, 200 μL of 0.50 mmol/L EDTA solution, 200 μL of 0.375 mmol/L nitrotetrazolium blue chloride (NBT) solution, and 200 μL of 0.50 mmol/L riboflavin solution were transferred to the same tube. The same procedure was performed for the control, in which phosphate buffer was used rather than the biosurfactant solution. The tubes were exposed to fluorescent light for 15 min with light dissipation using aluminum foil. Next, a 200-μL aliquot from each tube was transferred to a 96-well microplate to be read at 560 nm. The photochemical reduction inhibition capacity of NBT was calculated using the following equation:%I = [(Abs_0_ - Abs_1_)/Abs_0_ - Abs_BLANK_] × 100, in which Abs_BLANK_ corresponds to the same composition as the control without exposure to fluorescent light (Dasgupta, 2004).

### Evaluation of the Cytotoxic Potential of the Biosurfactant (MTT Assay)

The cytotoxic effect of the biosurfactant was assessed using the 3-[4,5-dimethyl-2-thiazolyl]-2,5-diphenyl-2-H-tetrazolium bromide (MTT) test (Alley et al., 1988; Mosmann, 1983). For such, the L929 (mice fibroblast) and RAW 264.7 (mice macrophage) cell lines were obtained from the Rio de Janeiro cell bank (Rio de Janeiro, Brazil) and kept in Dulbecco’s Modified Eagle’s Medium (DMEM) supplemented with 10% fetal bovine serum and 1% of an antibiotic solution (penicillin and streptomycin). The cells were kept at 37°C in a moist atmosphere enriched with 5% CO_2_. The L929 and RAW 264.7 cells (10^5^ cells/mL) were placed in 96-well plates containing DMEM medium and incubated for 24 h. Next, 10 μL of the biosurfactant solutions were added to the wells at a final concentration of 200 mg/L. Phosphate buffer (pH 7.4) at 150 mmol/L was used as the positive control and DMEM was the negative control. After 72 h of incubation, 25 μL of MTT (5 mg/mL) were added, followed by three more hours of incubation. The culture medium with the MTT was then aspirated and 100 μL of DMSO were added to each well. Absorbance was read in a microplate reader at a wavelength of 560 nm. The experiments were conducted in quadruplicate and the percentage of inhibition was calculated using GraphPad Prism version 7.0. An intensity scale was used for the determination of toxicity. Samples with inhibitory activity between 95 and 100% were considered toxic; those with inhibitory activity between 70 and 90% were considered moderately toxic and those with inhibitory activity less than 50% were considered non-toxic (Rodrigues et al., 2014).

### Study of Food Application of Biosurfactant

The biosurfactant was tested in a standard cookie formulation adapted from Zouari et al. (2016a; Table 1). The isolated biosurfactant was used in this formulation to partially (50%) and completely (100%) replace pasteurized egg yolk, generating two different types of dough (Formulations A and B) for the analysis of the physical and physicochemical properties.

**TABLE 1:** Composition of cookie dough formulations.

**Ingredients**	**Standard**	**Formulation**	**Formulation**
	**(%)**	**A (%)**	**B (%)**
White flour	47.0	47.0	47.0
Margarine	20.0	20.0	20.0
Sugar	15.0	15.0	15.0
Vanilla extract	3.0	3.0	3.0
Baking powder	1.0	1.0	1.0
Pasteurized egg white	10.0	10.0	10.0
Pasteurized egg yolk	4.0	2.0	0.0
Biosurfactant	0.0	2.0	4.0
Total	100.0	100.0	100.0

The dough was prepared using a modification of the method described by Zouari et al. (2016a). The ingredients were mixed in a blender (Arno Ciranda) for 7 min, followed by rolling and cutting into circular pieces measuring 50 mm in diameter. The pieces were baked for 5 min at 150°C, followed by an increase in temperature to 180°C for a further 15 min, with subsequent cooling, weighing, packaging, and storage at room temperature for 24 h.

### Properties and Energy Value of Cookies

The physical properties of the baked cookies (weight, diameter, thickness, and spreading factor) were analyzed as described by Noor-Aziah et al. (2012) and Zoulias et al. (2002). An analytical scale with a precision of 0.001 g (BEL Engineering) was used to determine the weight. To measure the diameter, four samples were selected at random and the total diameter was measured using digital calipers (Mtx). All four cookies were then rotated 90° and the new diameter was measured. The final diameter was expressed as the sum of the average of the two measurements of all cookies divided by four. Thickness was determined by stacking four cookies, measuring the total thickness and dividing by four. The scattering factor was obtained by dividing diameter by thickness.

The physicochemical properties of the cookies were determined based on the AOAC (2002). The moisture content was determined using the gravimetric method, considering the loss of weight of the samples submitted to heating in an oven at 105°C until reaching a constant weight. The total protein concentration was calculated using the Kjeldahl method, based on the acid digestion of organic matter followed by distillation, with nitrogen subsequently dosed by titration; the nitrogen value was then multiplied by a factor of 6.25. The gravimetric method was used for the determination of the fixed mineral residue (ash) based on the determination of the weight loss of the samples submitted to incineration at 550°C. The Bligh and Dyer (1959) cold extraction method was employed to quantify the lipid fraction, using a mixture of chloroform, methanol, and water. The energy value was determined by the sum of the carbohydrate, lipid, and protein values multiplied by 4, 9, and 4, respectively (Pires et al., 2017).

### Texture Profile Analysis

Before baking, the cookie dough was submitted to texture profile analysis (TPA) and the determination of firmness (resistance to breakage), cohesiveness and elasticity. The texture of the cookies after baking was evaluated using the compression test to determine firmness. For both tests, we used a Brookfield CT3 texture analyzer equipped with a 245 N load cell. The samples before and after cooking were compressed to 50% of their original height at a constant speed of 1 mm/s using a polymethyl methacrylate plate (width: 60 mm). For the TPA, a second compression was performed after an interval of 5 s, with firmness defined as the force at 50% of the sample height during the first compression. Cohesion was defined as the ratio between the compression work in the second compression cycle and the compression work in the first cycle. Elasticity was calculated using the relative height of the remaining sample when the initial force was recorded during the second compression (Zouari et al., 2016a).

### Statistical Analysis

The data were submitted to statistical analysis using the one-way procedure in Statistica^®^ (version 7.0), followed by a linear one-way analysis of variance (ANOVA). All triplicate results were expressed as mean ± standard deviation. Differences were examined using Tukey’s *post hoc* test, with a significance level of 95%.

## Results

### Biosurfactant Production

The prospect of producing yeast-based biosurfactants depends largely on identifying cheap, abundant raw materials. In this respect, waste streams constitute a potential source of substrates, which would offset the cost of waste treatment by the production of valuable co-products. Such nutrient sources can often be obtained with little or no cost (Borah et al., 2019). The biosurfactant produced by the yeast *S. cerevisiae* in a low-cost medium formulated with waste soybean oil and corn steep liquor was able to reduce the surface tension of the culture medium from 57.75 ± 0.20 to 26.64 ± 0.06 mN/m, with an interfacial tension against n-hexadecane of 9.12 ± 0.04 mN/m. Moreover, using ethyl acetate as the extraction solvent at a proportion of 1:4 (v/v) with the non-centrifuged medium, it was possible to isolate the biosurfactant with a yield of 5.84 ± 0.17 g/L.

### NMR and FT-IR Analysis

**FIGURE 1:**
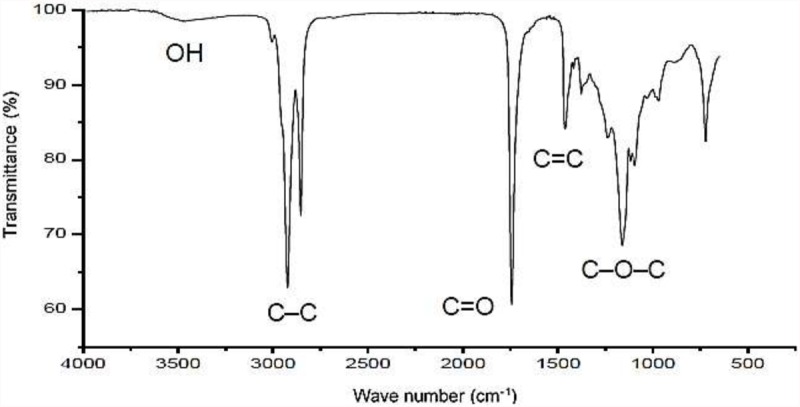
Infrared spectrum of biosurfactant produced by *S. cerevisiae* in medium supplemented with 1.0% waste soybean oil and 1.0% corn steep liquor.

**FIGURE 2:**
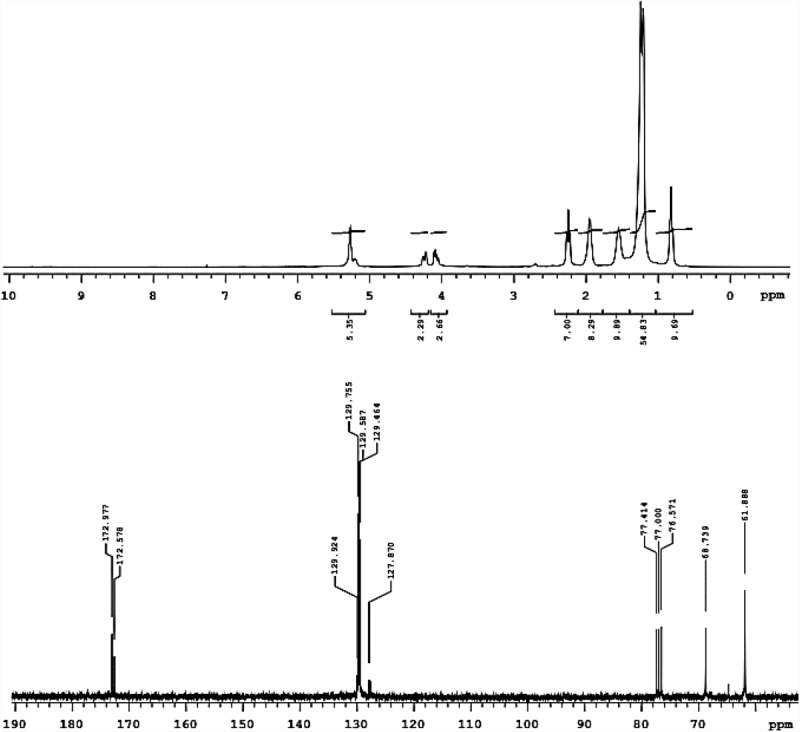
^1^H-NMR (above) and ^13^C-NMR (below) registered in deuterated chloroform of biosurfactant produced by *S. cerevisiae* in medium supplemented with 1.0% waste soybean oil and 1.0% corn steep liquor.

The infrared and NMR spectra of the isolated biosurfactant are displayed in Figures 1, 2, respectively. Figure 1 shows possible carbonyl groups (C=O) and single carbon bonds (C–C) corresponding to the respective stretches between 1500 and 2000 cm^–1^ as well as between 2700 and 3000 cm^–1^. Carbon double bonding (C=C) was also found in the region of approximately 1465 cm^–1^, as described by Nogueira et al. (2020) for the bioemulsifier from *Stenotrophomonas maltophilia* UCP 1601. A small stretching region was found in the 3000–3500 cm^–1^ range, indicating the presence of hydroxyl (OH) groups in the molecule. A considerable stretch was found at 1165 cm^–1^, indicating the existence of an ester group in the compound.

The ^1^H-NMR spectrum in Figure 2 shows the presence of methyl groups corresponding to signals between 0.7 and 0.9 ppm and a non-polar region of the molecule between 1.1 and 1.7 ppm. Bonds were also found between hydrogen and unsaturated carbon (1.8–2.1 ppm) as well as hydrogen and carbon neighbors to the unsaturated carbon (2.1–2.4 ppm). In addition to these signals, the presence of unsaturated carbon-bonded hydrogen was found between 5 and 5.5 ppm and a signal was found between 3.8 and 4.4 ppm, indicating the presence of a hydrogen neighbor to the oxygen of the ester-bonded molecule (Santos et al., 2017; Soares da Silva et al., 2017). This signal was confirmed in the carbon spectrum, which showed a characteristic signal of the ester group in a region less than 180 ppm (170–180 ppm). Thus, we can infer that the biosurfactant produced by *S. cerevisiae* was characterized as a glycolipid with ester linkages between fatty acids and carbohydrates in its structure.

### Fatty Acid Profile of Biosurfactant

**FIGURE 3:**
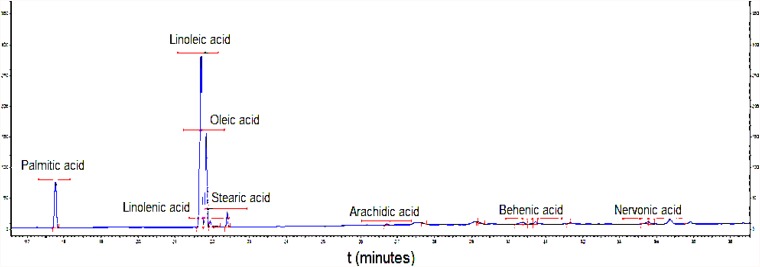
Chromatogram of fatty acid profile of biosurfactant produced by *S. cerevisiae* in medium supplemented with 1.0% waste soybean oil and 1.0% corn steep liquor.

Table 2 displays the composition of the fatty acids (Table 2) in the biosurfactant. Eight fatty acids were found in different proportions, with the absence of lauric, myristic, and eicoseinoic acid. The predominant fatty acids were linoleic acid (50.58 ± 0.25%) and oleic acid (28.79 ± 0.79%), with 18 carbons in their structures (Figure 3). Therefore, the biosurfactant has potential application in food formulations due to its nutritional value demonstrated by the high percentages of 18-carbon unsaturated fatty acids (oleic and linoleic acid) in its lipid portion (Burdge, 2019).

**TABLE 2:** Fatty acid profile of biosurfactant produced by *S. cerevisiae* in medium supplemented with 1.0% waste soybean oil and 1.0% corn steep liquor.

**Fatty acid**	**Composition (%)**
Palmitic acid (C16:0)	11.46 ± 0.04
Linoleic acid (C18:2)	50.58 ± 0.25
Oleic acid (C18:1)	28.79 ± 0.79
Linolenic acid (C18:3)	1.84 ± 0.06
Stearic acid (C18:0)	3.83 ± 0.03
Arachidic acid (C20:0)	0.41 ± 0.00
Behenic acid (C22:0)	0.91 ± 0.56
Nervonic acid (C24:0)	1.56 ± 0.77

### DSC and TG of Biosurfactant

**FIGURE 4:**
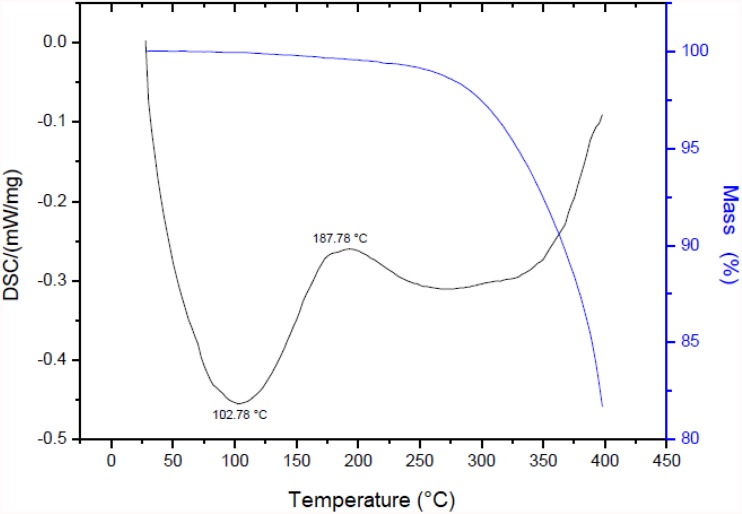
TGA and DSC of biosurfactant produced by *S. cerevisiae* in medium supplemented with 1.0% waste soybean oil and 1.0% corn steep liquor.

The thermograms of the biosurfactant produced by *S. cerevisiae* are displayed in Figure 4. The biosurfactant exhibited considerable thermal stability for its application in this work, with the loss of only 0.05% of its mass at a temperature of 102°C, 0.37% at 180.28°C and 0.43% at 200°C (blue curve). A significant decrease in mass was only found beginning at 250°C through to the final temperature (400°C), with mass loss in the range of 15.75%. As thermal degradation occurs when there is mass loss of approximately 5% (Kourmentza et al., 2018), the biosurfactant undergoes this type of degradation beginning at 330°C. The results of the DSC analysis showed an exothermic peak with a crystallization temperature (T_C_) of 102.78°C (onset temperature of 40.28°C) and an endothermic fusion peak of 187.78°C (onset temperature soon after the exothermic peak).

Therefore, if the biosurfactant is submitted to a baking process at a temperature of 180°C, it will not undergo a significant loss of mass, remaining stable and adequate for this application.

### Antioxidant Activity of Biosurfactant

Table 3 displays the percentage results of antioxidant activity measured by the sequestering of the DPPH and SOD organic radicals and the reduction in the phosphomolybdenum complex, visualized by the change in color of the solutions.

**TABLE 3:** Percentage of total antioxidant capacity (% TAC), DPPH radical sequestration (% I), and superoxide ion sequestration (% I) of different concentrations of biosurfactant from *S. cerevisiae*.

**Biosurfactant**	**% TAC**	**% I (DPPH)**	**% I**
**concentration**	**(ascorbic acid)**		**(superoxide ion)**
**(mg/mL)**			
40	–	21.35 ± 0.25	76.79 ± 5.29
20	212.70 ± 4.87	13.09 ± 0.62	76.31 ± 3.40
10	120.59 ± 0.12	11.37 ± 0.06	42.64 ± 0.62
5	77.34 ± 0.08	9.87 ± 0.32	18.75 ± 0.37
2.5	50.56 ± 1.27	8.48 ± 0.75	0.36 ± 0.00
1.25	30.62 ± 0.06	5.36 ± 0.12	–

According to the CAT assay, the biosurfactant has satisfactory antioxidant capacity, evidenced by the reduction of the phosphomolybdenum complex VI to V at a concentration of 5000 μg/mL. This reduction is remarkable, as evidenced by the color change from yellow to green, which was intensified with the increase in the biosurfactant concentration (Prieto et al., 1999). At lower concentrations (1250 μg/mL), the biosurfactant exhibited only 30% activity compared to a standard ascorbic acid solution (1000 μg/mL). In contrast, antioxidant activity greater than 200% was also found when using a concentration of 20000 μg/mL, indicating a linear relationship with the increase in the concentration of the biosurfactant.

Thus, the biosurfactant is a potential antioxidant at concentrations above 5000 μg/mL and can be applied in food formulations, as ascorbic acid is a recognized and highly used reducing agent.

In the DPPH assay, we evaluated the ability of the biosurfactant and two standards (Trolox and BHT) to prevent the oxidation of the DPPH radical by reducing it to hydrazine and consequently promoting a color change from purple to yellow, with a corresponding reduction in absorbance (Turnes et al., 2014). Analysing the results displayed in Table 3, considering a concentration of 1 mg/mL of the standards, the biosurfactant did not exhibit considerable antioxidant activity at concentrations below 20 mg/mL using the DPPH radical reduction method, with a maximum value 13.09 ± 0.62% compared to 88.84 ± 0.25% for Trolox and 83.37 ± 0.59% for BHT.

In the superoxide ion (O_2_^–^) sequestration assay, the biosurfactant exhibited a considerable inhibition percentage (greater than 42.64 ± 0.62%) at concentrations above 10000 μg/mL, evidenced by the visual color change from blue to yellow using the riboflavin-light-NBT system.

### Cytotoxic Potential of Biosurfactant

Biosurfactants are natural compounds that offer biocompatibility and low toxicity, making them strong candidates for the development of food products. However, there is a need to evaluate the toxicity of these biomolecules before proposing their application in food formulations.

In the cytotoxicity assay, the biosurfactant presented an inhibition rate of 14.78 and 9.90% for the non-cancerous strains RAW 264.7 and L929, respectively, when applied at a concentration of 200 μg/mL. In comparison, phosphate buffer (pH 7.4) presented an inhibition rate of 20.30 and 12.95% for RAW 264.7 and L929, respectively. According to Gomes Silva et al. (2017), inhibition rates of 1–20% indicate an absence of inhibitory activity. Therefore, the biosurfactant has no cytotoxic potential regarding the strains studied.

### Biosurfactant in Cookie Formulation

**FIGURE 5:**
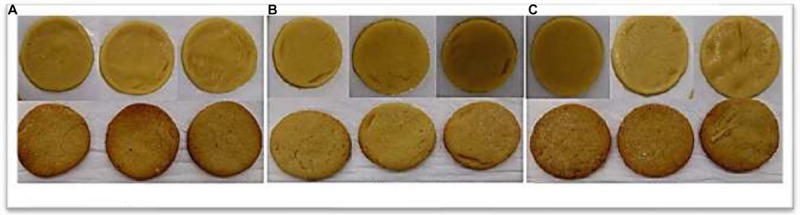
Cookies before and after baking. **(A)** standard formulation (4% egg yolk); **(B)** Formulation A (2% egg yolk and 2% biosurfactant); and **(C)** Formulation B (4% biosurfactant).

Figure 5 shows samples of cookies made from the standard formulation (4% egg yolk) and the partial (Formulation A – 2% egg yolk and 2% biosurfactant) and total (Formulation B – 4% biosurfactant) replacement of egg yolk with the biosurfactant before and after baking.

The mean results of the physical properties of the cookies (weight, diameter, thickness, and spreading factor) are shown in Table 4. The substitution of egg yolk with the biosurfactant had no significant effect, as all properties in the two formulations with different proportions of the biosurfactant were similar to those found with the standard formulation. Diameter was the only exception, which increased with the substitution in both formulations.

**TABLE 4:** Physical properties of cookies after baking for the Standard Formulation (4% egg yolk), Formulation A (2% egg yolk and 2% biosurfactant), and Formulation B (4% biosurfactant).

**Formulation**	**Weight (g)**	**Diameter**	**Thickness**	**Spread**
		**(mm)**	**(mm)**	**factor**
Standard	6.92 ± 0.71^a^	46.82 ± 0.50^a^	7.34 ± 0.24^a^	6.38 ± 0.05^a^
A	7.08 ± 0.57^a^	48.80 ± 0.45^b^	7.33 ± 0.45^a^	6.47 ± 0.31^a^
B	7.13 ± 0.66^a^	47.39 ± 0.22^ba^	7.64 ± 0.22^a^	6.20 ± 0.06^a^

*Different letters in same column denote significant differences (*p* ≤ 0.05, Tukey’s test).*

Regarding the physicochemical composition of the dough (Table 5), the moisture content changed significantly with both partial (Formulation A) and total (Formulation B) substitution. The protein content was also influenced by both substitutions, as lower values were found with the decrease in the egg yolk concentration. This can be attributed to the low concentration or absence of protein in the biosurfactant. On the other hand, the lipid content increased significantly with the increase in the biosurfactant concentration, proportionally influencing the energy value of the cookies.

**TABLE 5:** Physicochemical composition and energy value of cookies prepared with the Standard Formulation (4% egg yolk), Formulation A (2% egg yolk and 2% biosurfactant), and Formulation B (4% biosurfactant).

**Variable**	**Standard**	**Formulation**	**Formulation**
	**formulation**	**A**	**B**
Moisture (%)	0.07 ± 0.01^a^	0.10 ± 0.01^b^	0.03 ± 0.00^c^
Ash (%)	1.53 ± 0.11^a^	1.67 ± 0.08^a^	1.60 ± 0.01^a^
Lipids (%)	21.96 ± 0.79^a^	23.32 ± 0.60^ba^	25.10 ± 1.20^bc^
Proteins (%)	8.39 ± 0.04^a^	7.95 ± 0.13^b^	7.54 ± 0.08^c^
Carbohydrates (%)	68.05 ± 0.93^a^	66.95 ± 0.81^a^	65.73 ± 1.27^a^
Energy value (cal)	503.40 ± 3.58^a^	509.52 ± 2.65^ba^	518.95 ± 5.99^cb^

*Different letters on same line denote significant differences (*p* ≤ 0.05, Tukey’s test).*

In the TPA (Table 6), the partial (Formulation A) and the total (Formulation B) substitution of the yolk by the biosurfactant caused no significant change in the majority of variables analyzed. The only exception was firmness before baking with Formulation B (total substitution), which decreased considerably from 63.57 ± 2.84 N to 44.47 ± 6.40 N. This may have been due to the increased lipid concentration in the formulation with the addition of biosurfactant and removal of egg yolk.

**TABLE 6:** Texture profile analysis of dough before and after baking for the Standard Formulation (4% egg yolk), Formulaftion A (2% egg yolk and 2% biosurfactant), and Formulation B (4% biosurfactant).

**Formulation**	**Before baking**				**After baking Firmness (N)**
	**Firmness (N)**	**Cohesiveness**	**Elasticity (mm)**	**Adhesiveness (mJ)**	
Standard	63.57 ± 2.84^a^	0.70 ± 0.02^a^	0.77 ± 0.12^a^	1.67 ± 0.29^a^	445.59 ± 15.52^a^
A	66.18 ± 3.00^a^	0.72 ± 0.06^a^	0.77 ± 0.12^a^	1.17 ± 0.29^a^	427.88 ± 10.71^a^
B	44.47 ± 6.40^b^	0.62 ± 0.05^a^	0.83 ± 0.12^a^	1.50 ± 0.50^a^	426.60 ± 12.35^a^

*Different letters in same column denote significant differences (*p* ≤ 0.05, Tukey’s test).*

Overall, we can state that the partial and total replacement of egg yolk with the biosurfactant produced by *S. cerevisiae* did not negatively influence the characteristics of the final product, as the substituted formulations achieved similar results to the standard formulation. In addition, a simple evaluation of the aroma, taste, color, and texture revealed no significant differences between the formulations containing the biosurfactant and the standard formulation, with the latter exhibited a slightly darker color and greater firmness. To confirm these observations, future studies involving a complete sensory analysis will be conducted with trained tasters.

## Discussion

The characterization techniques employed in this study demonstrated that the isolated biosurfactant is a glycolipid that has a glycosidic bond between a sugar and the hydrophobic portion of the molecule as well as considerable potential for exploration and application (Salek and Euston, 2019). Other authors have reported the production of glycolipids by yeasts. In a study with 27 yeasts, Marcelino et al. (2019) reported the production of sophorolipids (type of glycolipid) in media with xylose and sugarcane bagasse, finding *Cutaneotrichosporon mucoides* UFMG-CM-Y6148 to be the best producer. Cultivating *Rhodotorula babjevae* Y-SL7 in a medium supplemented with various carbon sources, Guerfali et al. (2019) also found the production of glycolipids (determined by FT-IR analysis) with high emulsification indices and low toxicity. According to the authors, glycolipids are promising molecules for application in both the food and pharmaceutical industries, constituting a potential option for sustainable development in these sectors that may replace the synthetic compounds currently employed.

In a food application, it is preferable for the ingredients added to a formulation to have aggregate nutritional value, which makes the final product more attractive to consumers. This nutritional value is linked to the percentage of polyunsaturated fatty acids (PUFAs), i.e., the n-6 PUFA series [linolenic acid (LA)] and n-3 series [mainly alpha-linolenic acid (ALA), eicosapentaenoic acid (EPA) and docosahexaenoic acid (DHA)]. A greater occurrence of 18-carbon acids in the structure of ingredients increases the nutritional value, as these components are essential to the human organism (Burdge, 2019; Gramlich et al., 2019). As shown in Table 2, almost 80% of the fatty acids in the biosurfactant are PUFAs, with linoleic acid (C18: 2) accounting for the highest proportion (exceeding 50%). The benefits of ingesting these fatty acids in the diet for the prevention of cardiovascular disease are well described in the literature. Epidemiological studies also indicate that linoleic acid is associated with lower levels of plasma low-density lipoprotein cholesterol (LDL-C) and reduced liver fat and is inversely associated with the incidence of type 2 diabetes (Marangoni et al., 2020). Thus, the composition of the biosurfactant studied herein favors its use in food applications, as it is predominantly composed of linoleic and oleic acid, both of which have 18 carbons in the hydrophobic portion. The predominance of 18-carbon fatty acids in yeast glycolipids has recently been reported. Guerfali et al. (2019) and Souza et al. (2017) found high concentrations of oleic acid in biosurfactants isolated from *R. babjevae* Y-SL7 and *Wickerhamomyces anomalus* CCMA 0358, respectively.

The results of the thermal stability test indicate that the biosurfactant produced by *S. cerevisiae* is advantageous for industrial applications, since the DSC curve showed a relatively high melting peak, with stability in the temperature range tested (40–400°C) (Han et al., 2015). We also determined the change in mass due to thermal degradation using TG. This technique was adequate for the determination of the temperature at which thermal degradation of the biosurfactant occurs, demonstrating that for application in this case (maximum temperature of 180°C for baking cookies), the biosurfactant does not undergo significant mass loss, which would otherwise compromise the formulation.

There are no reports in the literature on the thermal stability of biosurfactants produced by *S. cerevisiae.* However, using thermal analysis on a biosurfactant produced by the yeast *Yarrowia lipolytica* MTCC9520, Radha et al. (2019) found mass loss greater than 9% at a temperature of approximately 220°C and 13.73% at a temperature of approximately 337°C. The DSC analysis of this same biosurfactant revealed crystallization and melting temperatures of 112.48 and 116.80°C, respectively, which is lower than the melting temperature found for the biosurfactant produced by *S. cerevisiae*. In a stability study of a rhamnolipid biosurfactant produced by *Burkholderia thailandensis*, Kourmentza et al. (2018) found a similar melting temperature (166.40°C). Arumugam and Shereen (2020) report a degradation temperature of 280°C for a rhamnolipid biosurfactant produced by *Enterobacter aerogenes*.

Antioxidant activity is a desirable property for substances to be incorporated in foods. According to Vecino et al. (2017), most biosurfactants contain a fatty acid chain that can prevent the generation of free radicals, acting as natural antioxidants. Thus, it is necessary to employ methods to determine the best type of action of the compound in question. In the present study, differences were found between the methods used for the determination of the antioxidant activity of the biosurfactant produced by *S. cerevisiae*, which can be explained by the principles and mechanisms of action specific to each method. Thus, we can infer that the biosurfactant studied presents better action in reducing complexes, as evidenced by the high percentage of total antioxidant capacity (212.70 ± 4.87%) referring to the biosurfactant concentration employed in the partial replacement of egg yolk in the cookies, followed by better action in superoxide ion inhibition by the sequestration mechanism. The presence of this mechanism in the biosurfactant is important, as the superoxide ion is toxic to the human metabolism, since it is produced during cellular respiration and has the ability to inactivate antioxidant enzymes, giving rise to other free radicals (Vadivel and Biesalski, 2011).

Regarding the results of DPPH sequestration, Merghni et al. (2017) obtained inhibition percentages of 74.6 and 77.3% using biosurfactants produced by species of *Lactobacillus*, which is approximately eight times higher than that obtained in the present investigation. Regarding total antioxidant capacity, a biosurfactant from marine *Streptomyces* sp. showed 80.475 ± 0.001% activity at a concentration of 200 μg/mL (Ramrajan et al., 2017). Takahashi et al. (2012), on the other hand, found that the mannosylerythritol lipids produced by the yeast *Pseudozyma hubeiensis* in a medium with soybean oil exhibited 50% DPPH radical scavenging activity at a concentration of 10 mg/mL and sequestration of superoxide ions greater than 50% at a concentration of less than 1 mg/mL. The results regarding the antioxidant activity of the biosurfactant from *S. cerevisiae* are promising and further tests should be conducted to confirm its antioxidant action and ensure another advantageous property for its application in food formulations.

Regarding the MTT method, the MTT molecule is reduced through mitochondrial succinate dehydrogenase activity when there is considerable cytotoxic potential in cells, resulting in formazan crystals that accumulate inside the cell and absorb light at a wavelength of 560 nm (Mosmann, 1983). In the present study, the MTT results reveal that the biosurfactant has potential food and/or cosmetic applications, as it exhibited no cytotoxicity at the concentration tested (200 μg/mL). However, further tests with other concentrations and other methods are necessary to ensure the safety of the biomolecule. The low toxicity of biological surfactants in relation to synthetic surfactants has previously been reported. Evaluating the cytotoxicity of a biosurfactant produced by *Rhodococcus* sp. 51T7 against mice 3T6 fibroblasts using this same method, Marques et al. (2009) obtained promising results for more restricted applications. Analyzing a baby hamster kidney cell line (BHK-21), Basit et al. (2018) found 63% cell survival using a concentration of 10^4^ μg/mL of the biosurfactant produced by *Bacillus cereus* MMC. A biosurfactant produced by *Lactobacillus helveticus* also exhibited no cytotoxic potential against the mouse fibroblast cell line ATCC L929 at concentrations of up to 25 × 10^3^ μg/mL (Sharma et al., 2014).

In the cookie formulations, the biosurfactant did not compromise the physical or physicochemical properties of the dough after baking, demonstrating that it is a good egg yolk substitute for reducing the amount of animal fat in foods. Despite the significant increase in the lipid content with the total replacement of the yolk, the presence of fatty acids in the hydrophobic structure of the biosurfactant favors this incorporation. Moreover, egg yolk, in addition to containing cholesterol, saturated fats, and triglycerides, can contain potentially toxic substances, such as trace elements and heavy metals, which, at high concentrations, can cause depression, hypertension, gastrointestinal cancer, and Alzheimer’s disease (Stadelman, 2003; Atamalekia et al., 2020). In contrast, a biosurfactant can have beneficial effects against cardiovascular disease, since it contains monounsaturated fat in its structure (Valenzuela et al., 2019). Despite the attractive properties of biosurfactants, the high production costs of these biomolecules remain a technological bottleneck, as is the case with most biotechnological products. On the other hand, the current consumer market is increasingly interested in the use of healthier food ingredients, which drives research and the optimization of processes for producing and extracting these biomolecules in order to make them more competitive as food additives.

Texture influences the intensity and perception of the sensory properties of food and is a determinant of acceptance on the part of consumers (Kiran et al., 2017). Since the biosurfactant had no significant effect on the texture profile of the dough after baking, it can be considered a potential ingredient for the food industry. It is necessary to perform this same test with other formulations to determine whether the biosurfactant can be incorporated without compromising the original texture of the food. Sensory evaluation tests should also be performed with the aim of broadening the applications of this biosurfactant.

Reports in the literature demonstrate that biosurfactants have the potential to improve the stabilization of salad emulsions and the texture profile of different types of flour-based foods, such as muffins and cookies. Biosurfactants can also be used to control consistency and solubilize flavoring oils in bakery products and ice cream. Adding a biosurfactant produced by *Bacillus subtilis* SPB1 at concentrations above 0.5% to a cookie formulation, Zouari et al. (2016a) found changes in the dough texture profile, with significant reductions in firmness and elasticity (*p* ≤ 0.05) and greater cohesion. Kiran et al. (2017) incorporated a lipopeptide produced by the marine bacterium *Nesterenkonia* sp. into a muffin formulation at a concentration of 0.75% and found enhanced smoothness of the end product due to increased elasticity and cohesion as well as decreased firmness. Adding a biosurfactant produced by *Candida utilis* UFPEDA 1009 at a concentration of 0.7% to different salad dressing formulations containing Guar gum and carboxymethyl cellulose (CMC), Campos et al. (2019) found greater stability and firmness after 30 days of storage, considering the biosurfactant to be a good emulsifier.

Further studies are needed to promote the use of biosurfactants in the food sector, seeking greater economic viability and less waste generation, since replacing only the egg yolk at the concentrations evaluated is not yet economically viable. However, the results showed that the biosurfactant can perform functions similar to those of the ingredients commonly used in food and can even entirely replace egg.

## Conclusion

The present findings demonstrate that a glycolipid biosurfactant can be produced by the yeast *S. cerevisiae* grown in a medium with agro-industrial waste products with satisfactory yield using the isolation methodology employed. The biosurfactant is non-toxic, which suggests its safe use, and has considerable antioxidant activity. Its thermostability demonstrates that it can be employed in production systems that use relatively high temperatures. Moreover, the complete replacement of egg yolk by the biosurfactant shows promising results in relation to the physical, physicochemical, and textural properties of cookies. Further studies are needed to make production on the industrial scale economically viable and reduce the concentration used in this type of formulation. Based on the positive results obtained in this initial study, this microbial surfactant has biotechnological potential for the food industry.

## Data Availability Statement

The raw data supporting the conclusions of this article will be made available by the authors, without undue reservation, to any qualified researcher.

## Author Contributions

LS conceived and designed the experiments. BR performed the experiments. JG analyzed the data and contributed to the analysis. BR and LS wrote the manuscript. All authors contributed to this work.

## Conflict of Interest

The authors declare that the research was conducted in the absence of any commercial or financial relationships that could be construed as a potential conflict of interest.
